# Stroke risk in patients with device-detected atrial high-rate episodes

**DOI:** 10.1007/s12471-017-1047-3

**Published:** 2017-10-20

**Authors:** Ö. Erküner, M. Rienstra, I. C. Van Gelder, U. Schotten, H. J. G. M. Crijns, J. G. L. M. Luermans

**Affiliations:** 1 Department of Cardiology, Maastricht University Medical Center +, Maastricht, The Netherlands; 2 Cardiovascular Research Institute Maastricht (CARIM), Maastricht University, Maastricht, The Netherlands; 3 Department of Cardiology, Thorax Center, University of Groningen, University Medical Center Groningen, Groningen, The Netherlands; 4 Department of Physiology, Maastricht University, Maastricht, The Netherlands

**Keywords:** Atrial high-rate episode, Stroke, Atrial fibrillation, Cardiovascular implantable electronic device, Antithrombotic therapy

## Abstract

Cardiovascular implantable electronic devices (CIEDs) can detect atrial arrhythmias, i. e. atrial high-rate episodes (AHRE). The thrombo-embolic risk in patients showing AHRE appears to be lower than in patients with clinical atrial fibrillation (AF) and it is unclear whether the former will benefit from oral anticoagulants. Based on currently available evidence, it seems reasonable to consider antithrombotic therapy in patients without documented AF showing AHRE >24 hours and a CHA_2_DS_2_-VASc score (congestive heart failure, hypertension, age ≥75 years [doubled], diabetes mellitus, prior stroke [doubled], vascular disease, age 65–74 years and female sex) ≥1, awaiting definite answers from ongoing randomised clinical trials. In patients with AHRE <24 hours, current literature does not support starting oral anticoagulation. In these patients, intensifying CIED read-outs can be considered to find progression in AHRE duration sooner, enhancing timely stroke prevention. The notion that AHRE and stroke coincide perseveres but should be abandoned since CIED data show a clear disconnect.

## Introduction

Cardiovascular implantable electronic devices (CIEDs) with an atrial lead can detect episodes of atrial arrhythmias, regardless of the presence of symptoms. Device-detected atrial high-rate episodes (AHRE), in the absence of symptoms referred to as subclinical atrial tachy-arrhythmias, are actually quite common. The incidence of AHRE in patients without a history of atrial fibrillation (AF) is approximately 25% after 1 year and 35% after 2 years of follow-up [1–3]. For patients with a history of AF, the incidence of AHRE is approximately 56–71% after 1 year [4–6].

AHRE differ from clinical AF in the mode of documentation, i. e. clinical AF is ascertained on an electrocardiogram, whereas AHRE are solely recorded on a CIED read-out [7]. Furthermore, AHRE and AF differ regarding thrombo-embolic risk. Clinical AF is associated with an increased risk of thrombo-embolism depending on the presence of risk factors, i. e. the CHA_2_DS_2_-VASc score (congestive heart failure, hypertension, age ≥75 years [doubled], diabetes mellitus, prior stroke [doubled], vascular disease, age 65–74 years and female sex) [8]. In AHRE patients, however, the thrombo-embolic risk appears to be lower than in clinical AF [2, 6, 9–11]. The lower thrombo-embolic event rate could be caused by the fact that AHRE are viewed as one entity, whilst there may be different types of AHRE. AHRE with a lower mean atrial rate, i. e. <300 beats per minute (bpm), may not represent AF but rather an atrial tachycardia, which confers a lower thrombo-embolic risk [12], whereas AHRE with a mean atrial rate >300 bpm might more robustly represent AF or atrial flutter, leading to an increased thrombo-embolic risk [13].

Current guidelines recommend starting anticoagulant therapy in AF patients with a CHA_2_DS_2_-VASc score ≥2 and considering antithrombotic therapy in patients with a CHA_2_DS_2_-VASc score of 1 [7, 14]. For AHRE, however, no recommendations regarding antithrombotic therapy are made in the guidelines, largely because of lack of evidence for any benefit of antithrombotic treatment in patients with AHRE [7, 14].

In this point of view paper, we summarise the evidence regarding thrombo-embolic risk in patients with AHRE, elaborating on the duration of AHRE and on the temporal relationship of AHRE and thrombo-embolism. To conclude, we propose a flowchart for antithrombotic management of these patients.

## Current evidence

### Duration of AHRE and thrombo-embolic risk in patients without a history of atrial fibrillation

The Asymptomatic Atrial Fibrillation and Stroke Evaluation in Pacemaker Patients and the Atrial Fibrillation Reduction Atrial Pacing Trial (ASSERT) is the only large, prospective trial to assess AHRE and thrombo-embolism in patients without a history of clinical AF [2]. In this study, 2,580 patients with a recently implanted pacemaker or defibrillator were included. All patients were 65 years of age or older and had hypertension. The devices of these patients were interrogated at regular six-monthly intervals in order to detect AHRE, which was defined as an atrial rate of at least 190 bpm lasting for at least 6 minutes. All AHRE were blindly judged. Three months after inclusion, 261 patients (10.1%) already showed at least one atrial high-rate episode. After a mean follow-up of 2.5 years, this was the case in 34.7% of the patients.

During the follow-up period of the ASSERT study, a stroke or systemic embolism occurred in 11 of 261 patients (4.2%) in whom AHRE was detected within 3 months after inclusion, compared to 40 of the remaining 2,319 patients (1.7%). This translates into an annual thrombo-embolic event rate of 1.7% in patients with AHRE within 3 months after inclusion, compared to 0.7% in patients who did not show AHRE within 3 months after inclusion (hazard ratio [HR] 2.49; 95% confidence interval [CI], 1.28–4.85; *p* = 0.007). In this analysis however, the 633 patients who developed AHRE after the initial monitoring period of 3 months were included in the control group of 2,319 patients, possibly leading to a higher thrombo-embolic rate in this group.

In a subanalysis of the ASSERT study regarding the duration of AHRE and thrombo-embolic risk, all AHRE during monitoring and follow-up were taken into account, irrespective of the time of occurrence [15]. In a time-dependent Cox regression model, the thrombo-embolic risk only increased in patients showing AHRE >24 hours (HR 3.24, 95% CI 1.51–6.95, *p* = 0.003 compared to patients without AHRE). For AHRE lasting <24 hours, the thrombo-embolic risk seems to be comparable to patients without AHRE. Even in patients showing 6–24 hours of AHRE, the thrombo-embolic risk was not increased (HR 1.32, 95% CI 0.40–4.37, *p* = 0.646). In addition, the number of AHRE did not affect thrombo-embolic risk [2].

### Duration of AHRE and thrombo-embolic risk in patients with a history of atrial fibrillation

In the remainder of the studies regarding AHRE, patients with a history of AF were also included, ranging from 20% of the patients in TRENDS to all included patients in the Italian AT500 Registry (Tab. 1; [4–6, 16]). Therefore, the sole effect of AHRE on thrombo-embolic risk cannot be reliably assessed based on these studies.

**Table 1:** Overview of trials regarding AHRE and stroke rates

Trial	*n*	Prior AF (%)	Mean CHADS_2_	Prior OAC (%)	Definition of AHRE		AHRE + annual TE (%)	AHRE − annual TE (%)	RR for TE	*p*
					Atrial rate	Duration				
ASSERT [2]	2,850	0	2.2	7.5	>190 bpm	>6 min	1.7	0.7	2.5	0.007
TRENDS [6]	2,486	20	2.2	20.8	>175 bpm	≥5.5 h	2.4	1.1	2.2	0.06
Turakhia et al. [16]	9,850	41	3.2	5.4	AT/AF	≥5.5 h	–	–	4.2	<0.05
MOST [15]	312	60	–	–	>220 bpm	>5 min	–	–	2.8^a^	0.001
AT500 [4]	725	100	–	36.4	AT/AF	>24 h	–	–	3.1	0.044

*AF* atrial fibrillation, *AHRE* atrial high-rate events, *AT* atrial tachycardia, *CHADS*_*2*_ congestive heart failure, hypertension, age ≥75 years, diabetes mellitus and prior stroke (doubled), *OAC* oral anticoagulation, *bpm* beats per minute, *RR* relative risk, *TE* thrombo-embolism

^a^ Combined endpoint of death and non-fatal stroke

In the TRENDS trial, 2,486 patients with a CIED and a CHADS_2_ (congestive heart failure, hypertension, age ≥75 years, diabetes mellitus and prior stroke [doubled]) score ≥1 were analysed [6]. Mean age was 71 years in this population and 498 patients (20%) had a history of AF. Atrial tachycardia (AT)/AF, defined as an atrial rate >175 bpm lasting ≥20 seconds, was observed in 47% of these patients during an average follow-up of 1.4 years. When patients were divided in groups based on the maximum AT/AF burden on any day during follow-up, the annual thrombo-embolic risk tended to double in patients with a high AT/AF burden of ≥5.5 hours per day, compared to patients with zero or low burden, i. e. <5.5 hours per day (2.4% vs. 1.1% per year; HR 2.20, 95% CI 0.96–5.05, *p* = 0.06) [6]. The annual thrombo-embolic event rates in the AHRE populations were lower than in AF patients with a comparable risk profile [8].

In the Italian AT500 Registry [4], 725 patients with atrial fibrillation and a pacemaker with a mean age of 71 years were included. Device-detected AF episodes were defined as ≥5 minutes in duration, no minimally required atrial rate was reported. AF episodes of >24 hours were associated with an increased risk of thrombo-embolism after a median follow-up of 22 months (HR 3.1, 95% CI 1.1–10.5, *p* = 0.044; no percentages reported for patients with and without AHRE). Botto et al. [5] further elaborated on this finding by combining the duration of device-detected AF with the CHADS_2_ score. This way, two subpopulations with a significant difference in annual thrombo-embolic risk were determined (0.8% vs. 5%, *p* = 0.035). With increasing CHADS_2_ scores, a decreasing duration of AHRE is sufficient to provoke a high risk of stroke [5].

In a subgroup analysis of the MOde Selection Trial (MOST) [16], 312 patients with a median age of 74 years were included, of whom 60% had a history of supraventricular arrhythmias. AHRE was defined as an atrial rate >220 bpm lasting >5 minutes and was present in 160 (51.3%) patients over a follow-up of 27 months. These patients had an increased risk of death or nonfatal stroke when compared with those without AHRE (20.6% vs. 10.5%; HR 2.79, 95% CI 1.51–5.15, *p* = 0.001; no annual rates reported) [16]. This rate is higher than in the studies discussed above, most likely due to the population of the MOST being older and having more comorbidities.

### Temporal relationship of AHRE and thrombo-embolism

The classical pathophysiological idea is that atrial fibrillation causes thrombo-embolism via mechanical stasis in the atrium, leading to clot formation. In this respect, it seems logical that a higher AHRE or AF burden is associated with a higher thrombo-embolic risk, which has been shown in the ASSERT trial. In this study, an AHRE of ≥24 hours compared to AHRE <24 hours or no AHRE at all was associated with thrombo-embolism within 30 days [15]. Similarly, Turakhia et al. have shown in a Veterans Administration population with stroke that patients showed more often AHRE ≥5.5 hours in the 30 days preceding the stroke compared with a control period of days 91 to 120 prior to the stroke in the same patients (HR 4.2, 95% CI 1.5–13.4) [17].

**Fig. 1:**
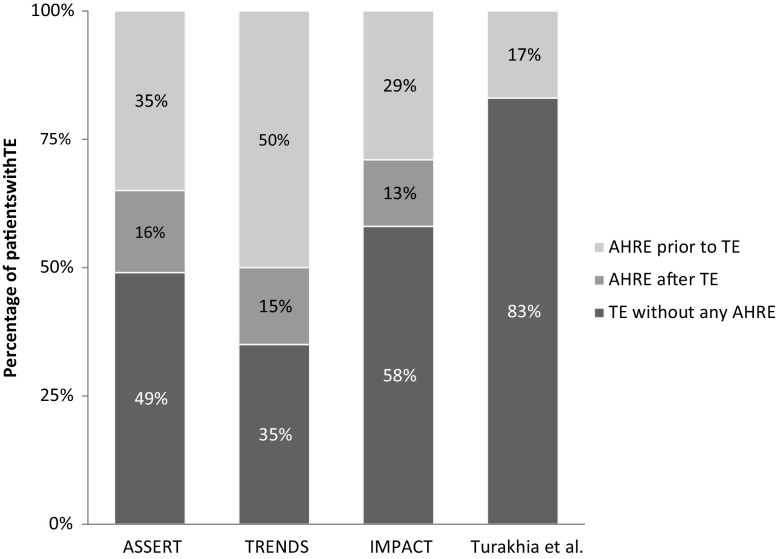
Temporal Relationship between AHRE and TE. The stacked bar chart depicts the patients with a TE in the respective study. The percentages represent the proportion of patients who showed AHRE before TE, after TE, and the patients who did not show any AHRE at all. (*AHRE* atrial high-rate episodes, *TE* thrombo-embolism)

In contrast to the classical pathophysiology of thrombo-embolism in AF however, a clear temporal relationship cannot be well established in a substantial proportion of patients with AHRE and thrombo-embolism. That is to say, thrombo-embolism is not preceded by AHRE or AF, but the arrhythmia rather occurs after the thrombo-embolic event. This has been studied in subanalyses of both the ASSERT and TRENDS study. Brambatti et al. [18] showed that only 26 of the 51 patients (51%) with a thrombo-embolic event during follow-up in the ASSERT study showed AHRE, of which 8 patients (30.8%) only showed AHRE after the thrombo-embolic event. Daoud et al. [19] showed similarly that in only 20 (50%) of the 40 patients with thrombo-embolism in the TRENDS study, AT/AF was detected prior to the event, nine of whom did not show any AT/AF in the 30 days prior to the event (Fig. 1).

Furthermore, in the IMPACT trial [20], 2,718 CIED patients were randomised to usual office-based follow-up with anticoagulation determined by standard clinical criteria or starting and stopping anticoagulation based on remote rhythm monitoring. AHRE was defined as ≥36 of 48 atrial beats with an atrial rate ≥200 bpm. Of the included patients, 330 (12.1%) had a history of AF or atrial flutter. The compared antithrombotic therapy strategies did not show any differences in ischaemic stroke (0.7 vs. 1.3 per 100 patient-years; HR 0.55, 95% CI 0.23–1.34, *p* = 0.188), even though there was an association between AHRE burden and thrombo-embolism [20]. However, there are some limitations to the IMPACT study, i. e. the low AHRE rate, the predominant use of vitamin K antagonists as anticoagulation therapy, the time to adequate INR (international normalised ratio), and the strategy of discontinuation and re-initiating oral anticoagulants based on the presence/absence of AHRE.

**Fig. 2:**
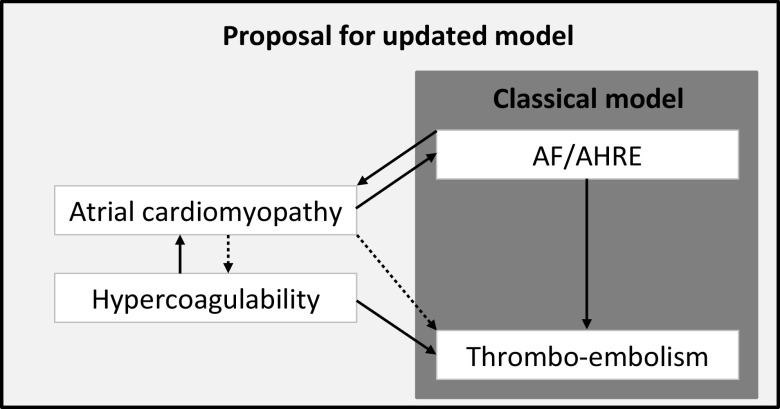
Proposed updated model of TE in AF/AHRE. The figure depicts the classical model of thrombo-embolic stroke in AF and AHRE and a proposal for an updated model. In this proposal, atrial cardiomyopathy can lead to TE through hypercoagulability and hypercoagulability can lead to AF/AHRE through atrial cardiomyopathy. (*TE* thrombo-embolism, *AF* atrial fibrillation, *AHRE* atrial high-rate episodes)

All together, these findings suggest that AHRE/AF and stroke do not always occur in the classical pathophysiological order. To address this issue, Kamel et al. [21] proposed an updated model for thrombo-embolic stroke. In this model, atrial cardiomyopathy plays a key role and can result in both AHRE/AF and in thrombo-embolism, explaining the situations in which thrombo-embolism precedes the occurrence of AHRE/AF. Ageing and systemic vascular risk factors can cause an abnormal atrial substrate leading to atrial cardiomyopathy, which can be characterised by atrial dilatation, mechanical dysfunction, fibrosis, and/or endothelial dysfunction [21]. Another factor causing an abnormal atrial substrate might be hypercoagulability, the increased potential of blood or plasma to generate thrombin and fibrin [22]. Spronk et al. have shown that hypercoagulability can induce atrial fibrosis and lead to a substrate for AF [22]. Similarly, the increased risk of thrombo-embolism in patients with atrial cardiomyopathy might be mediated by hypercoagulability (Fig. 2).

In addition to the temporal disconnect of AHRE and thrombo-embolism observed in the trials described above, some patients even developed a stroke without showing any AHRE at all. In ASSERT, 25 (49%) of the 51 patients with a stroke did not show any AHRE, in TRENDS this was the case in 20 (50%) of 40 patients, in IMPACT in 40 (58%) of 69 patients, and in the study by Turakhia et al. in 156 (83%) of 187 patients (Fig. 1). These strokes were either the result of other pathophysiological mechanisms, i. e. cardiac embolism not related to AHRE/AF, lacunar infarction, arterial dissection, and atherosclerosis or perhaps due to atrial cardiomyopathy in an early phase, with no manifestation of AF/AHRE yet, compatible with the model as presented in Fig. 2.

### When to start antithrombotic therapy in patients with AHRE without a history of atrial fibrillation

**Fig. 3:**
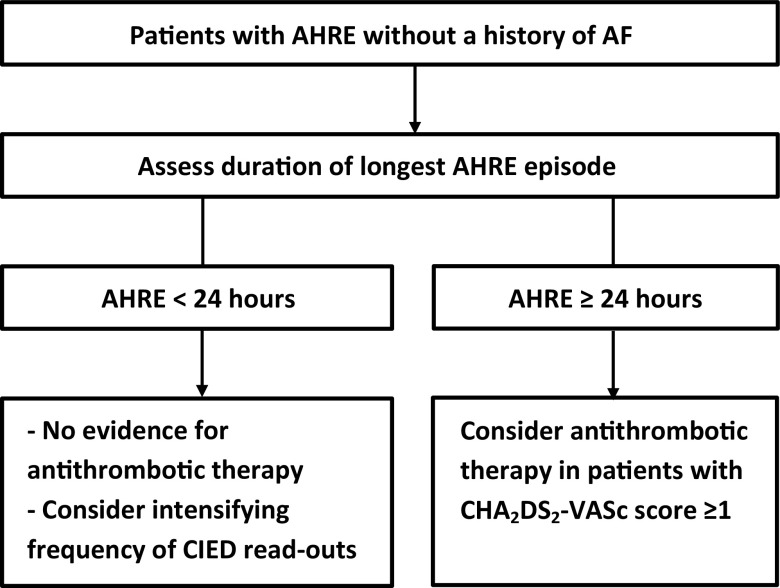
Recommendations for patients showing AHRE. The figure is a proposed flowchart for the antithrombotic therapy of patients with atrial high-rate episodes. (*AF* atrial fibrillation, *AHRE* atrial high-rate episodes, *CHA*_*2*_ *DS*_*2*_ *-VASc* congestive heart failure, hypertension, age ≥75 years (doubled), diabetes mellitus, prior stroke (doubled), vascular disease, age 65–74 years and female sex, *CIED* cardiac implantable electronic device)

Taking the available literature into consideration, it seems reasonable to consider antithrombotic therapy in patients without documented AF showing AHRE >24 hours and a CHA_2_DS_2_-VASc score ≥1, awaiting definite answers from ongoing randomised clinical trials (Fig. 3). In patients with shorter AHRE, current evidence does not support starting antithrombotic therapy in the absence of clinical AF. Duration should be the only characteristic of AHRE to take into consideration before deciding to start antithrombotic therapy, since the timing or number of AHRE do not seem to be useful in identifying patients at high risk for thrombo-embolism. For patients with <24 hours of AHRE, one could consider intensifying CIED read-outs in order to find a progression in AHRE relatively early. As a result, the delay to starting antithrombotic therapy can be reduced. As for the cut-off point of the CHA_2_DS_2_-VASc score, considering antithrombotic therapy in patients with a score of ≥1, analogous to clinical AF patients, seems reasonable, even though the CHA_2_DS_2_-VASc score has not been validated in an AHRE cohort.

Currently recruiting trials ARTESiA (Apixaban for the Reduction of Thrombo-Embolism in patients with Device-Detected Sub-Clinical Atrial fibrillation, ClinicalTrials.gov Identifier: NCT01938248) and NOAH-AFNET 6 (Non-vitamin K Antagonist Oral Anticoagulants in Patients With Atrial High Rate Episodes—Atrial Fibrillation NETwork 6, ClinicalTrials.gov Identifier: NCT02618577) will give us further insights in the role of antithrombotic therapy in patients with AHRE without a history of atrial fibrillation. In these prospective, parallel-group, randomised, double-blind trials, patients with AHRE will receive either placebo or a non-vitamin K antagonist oral anticoagulant, apixaban and edoxaban, respectively. In ARTESiA, AHRE are defined as an atrial rate of >175 bpm lasting ≥6 minutes, whereas in NOAH-AFNET 6 the definition is slightly different, i. e. an atrial rate of ≥180 bpm and ≥6 minutes in duration.

## Conclusion

AHRE are a common finding in patients with pacemakers and defibrillators without a history of atrial fibrillation. AHRE are associated with an increased thrombo-embolic risk, albeit lower than in clinical AF, in part depending on the duration of the AHRE. Based on currently sparse literature, it seems reasonable to start antithrombotic therapy in patients without clinical AF with a CHA_2_DS_2_-VASc score ≥1 in whom at least one episode of the AHRE lasting >24 hours has been detected, irrespective of the timing and number of AHRE, awaiting definite answers from ongoing randomised clinical trials. In patients with shorter AHRE, current evidence to start antithrombotic therapy in the absence of clinical AF is lacking.
